# Pattern and outcomes of paediatric medical admissions at the Living Word Mission Hospital, Aba, South East Nigeria

**DOI:** 10.11604/pamj.2018.30.202.15966

**Published:** 2018-07-10

**Authors:** Nneka Chioma Okoronkwo, Chukwuemeka Ngozi Onyearugha, Chioma Akunnaya Ohanenye

**Affiliations:** 1 Department of Paediatrics, Abia State University Teaching Hospital Aba, Abia State

**Keywords:** Pattern, outcome, admissions, paediatrics, Mission Hospital, Nigeria

## Abstract

**Introduction:**

There is a decline in child mortality rate globally, courtesy of the erstwhile Millennium Development Goals. However, under-five mortality is still high in the African sub-regions. The need to review the morbidity and mortality pattern among children admitted into private health settings, where 60% of the medical conditions of the masses are being attended to in the sub-regions, cannot be overemphasized. This study aimed at documenting the morbidity pattern and outcomes of admissions among children admitted into the Living Word Mission Hospital (LWMH), Aba, Nigeria.

**Methods:**

This was a retrospective descriptive study over a 3 year period. The study population comprised of all children aged 1 month to 15 years that were admitted into the pediatric wards of the Living Word Mission Hospital, Aba, Nigeria. The age, gender, diagnoses and disease outcome of these patients, were all retrieved from the pediatric ward registers and hospital medical records. The data were analyzed using SPSS, version 20.0.

**Results:**

There were 2278 pediatric medical cases admitted over the study period. Males were 1364 and females were 914, giving a male: female ratio of 1.5:1. More than 90% of these patients were aged < less than 5 years. Severe malaria (31.1%), septicaemia (16.6%), bronchopneumonia (15.4%), uncomplicated malaria (11.9%), acute watery diarrhea (10.5%) and meningitis (3.7%) were the leading causes of admission. Mortality rate was 5.7%, with 87.5% of these deaths occurring in under-fives. Septicaemia (34.6%) and Severe malaria (23.2%) were the leading causes of death.

**Conclusion:**

There is a high rate of paediatric admissions at Living Word Hospital, Aba. The under-five population remains a vulnerable group to both childhood morbidity and mortality. Septicaemia, malaria, bronchopnuemonia and acute watery diarrhoea were the leading causes of morbidity and mortality. Childhood mortality at LWMH is lower than observed in most government hospitals in Nigeria.

## Introduction

Infections and communicable diseases have remained top causes of childhood morbidity and mortality in Africa [1,2]. It is a great concern that despite the preventable nature of the causes of childhood deaths in Africa, childhood mortality rate is still high in our sub-region [3]. The global under-five (U-5) mortality rate decreased from 91 deaths per 1000 live births in 1990 to 43 deaths per 1000 live births in 2015 [4]. This decline is courtesy of the erstwhile MDG [4,5]. However, U-5 mortality is still high in the African sub-regions, with Nigeria losing 2,300 children aged less than 5 years to death every day [6]. This has made Nigeria the second largest contributor to global U-5 mortality, according to UNICEF [6]. It is estimated that up to 60% of the medical conditions of the masses are attended to by the private health sector in Nigeria [7]. Again, the recurrent “strike actions” in the government health sector has made the private health sector more relevant to the health needs of the people in Nigeria [8]. The mission hospitals are very important part of the private health sector, especially in south eastern Nigeria [8]. National medical data for making public health policies cannot be complete without accounting for the statistics from the private health sectors, especially in a country like Nigeria. Previous studies have documented childhood morbidity and mortality in various health institutions across Nigeria [9-13]. However, these studies were conducted in government owned health institutions. The need to review the pattern of childhood morbidity and mortality in the private health sector cannot be over-emphasized. This study was therefore aimed at determining the pattern of morbidity and mortality among children admitted to the Living Word Mission Hospital Aba, Abia State, Nigeria.

## Methods

Living Word Mission Hospital (LWMH) Aba is a Christian Mission Hospital founded in 1996 by The Living Word Ministries International. It caters for both adults and children. Scope of care includes both medical and surgical cases. The paediatrics department of this hospital started in 2002. This department is manned by 4 medical officers, one part time senior registrar and a visiting consultant pediatrician. The consultant paediatrician consults once a week, does ward round once every week and is also available during calls or when the junior doctors have difficulty in managing any patient. It is the biggest private hospital in Aba. Its location at the centre of the town attract a reasonable patient load to the hospital. It attends to approximately 1000 paediatric patients per year [14]. This study was a retrospective review of medical records over a 3 year period. The details of all patients that are admitted into the paediatrics department of the LWMH Aba are recorded in hard copy patient files which are kept with the hospital medical records department. Permission was obtained from the Chief Medical Director of the LWMH, Aba, before commencing the study. The total number of all the patients, aged 1 month to 15 years that were admitted into the emergency room and children's ward of LWMH between January 2012 and December 2014 were retrieved from the hospital's medical records. Information extracted were the following: age and sex of the patient, presenting complaints, investigation results and diagnoses, duration of admission and outcome of illness. After data collation and cleaning, data were analysed using SPSS (Statistical Package for the Social Sciences) software, version 20.0 [15]. Frequency tables and percentages were generated for all the major variables of interest. Categorical variables were presented as percentages, pie and bar charts, while comparisons between such variables were done using the Pearson Chi Square test. A confidence interval of 95% was used and for all analyses a p-value < 0.05 was taken as statistically significant.

## Results

**Figure 1:**
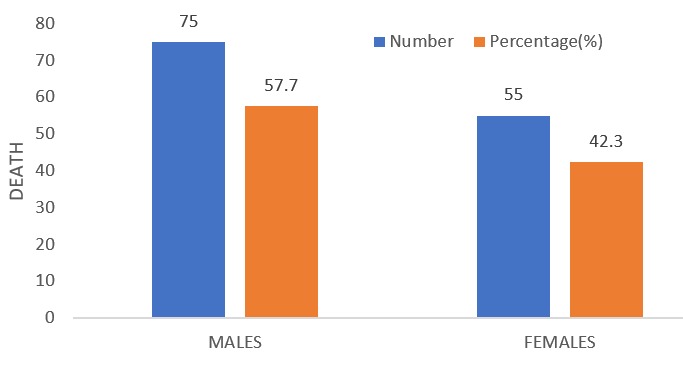
Sex distribution of the patients that died

**Figure 2:**
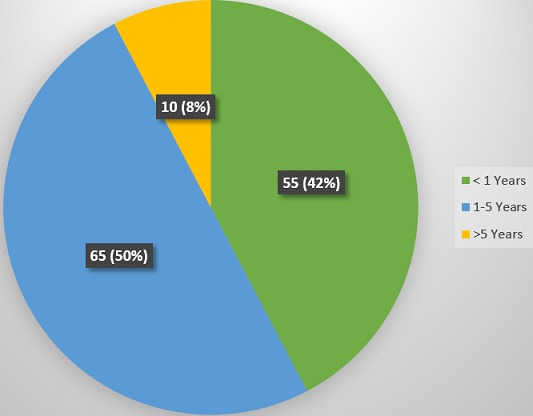
Age distribution of the patients that died

**Figure 3:**
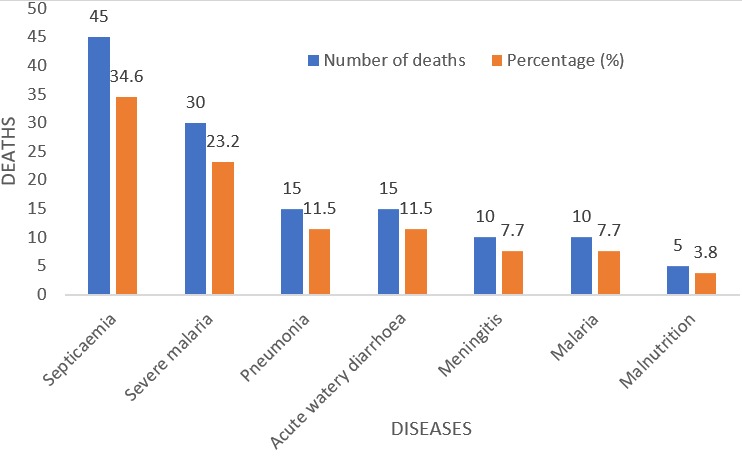
Diagnostic causes of death

There was a total of 2350 paediatric cases (both surgical and medical) admitted over the study period out of which 2278 were medical cases. For the medical cases, males were 1364 and females were 914, giving a male: female ratio of 1.5:1. More than 90% of these patients were aged < less than 5 years (**Table 1**). Uncomplicated malaria, severe malaria, septicaemia, pnuemonia, acute watery diarrhoea and meningitis were the leading morbidities recorded over the study period (Table 2). One hundred and thirty (130) of these patients died, giving a mortality rate of 5.7%. Fifty three (53) were discharged against medical advice, 2062 were well and discharged, 33 were referred and none absconded (Table 3). Fifty eight percent (57.7%) of this death occurred in the male patients while 42.3% were recorded among the females (Figure 1). There was a statistical significant association between death and sex (p-value = 0.031). More than 90% of these deaths occurred in the U-5s, out of which 42.3% involved infants less than 1 year (Figure 2). However, there was no statistical significant association between death and age (p-value = 1.365). Septicaemia, severe malaria, pnuemonia and acute watery diarrhoea were the major diseases causing death in the study population (Figure 3).

**Table 1:** Sex and age distribution of the study population

	Sex		
Age (Years)	Male n (%)	Female n (%)	Total n (%)
< 1	518 (22.7)	330 (14.5)	848 (37.2)
1-5	636(27.9)	432 (19.0)	1068 (46.9)
>5	210 (9.2)	152 (6.7)	362 (15.9)
**Total**	1364 (59.9)	914 (40.1)	2278 (100.0)

**Table 2:** Diagnosis on admission over the study period

Diseases	Frequency (n)	Percentage (%)
Severe malaria	709	31.1
Septicaemia	378	16.6
Pneumonia	350	15.4
Uncomplicated Malaria	271	11.9
Acute watery diarrhoea	240	10.5
Meningitis	84	3.7
Seizures Disorders	29	1.3
Sickle Cell Crises	28	1.3
Pharyngitis	22	1.0
Acute kidney Injury	11	0.5
Measles	16	0.7
Acute Severe Asthma	16	0.7
Malnutrition	14	0.6
Peptic Ulcer Disease	12	0.5
Hepatitis	10	0.4
Adenoiditis	10	0.4
Shigellosis	10	0.4
Others	68	3.0
**TOTAL**	**2278**	**100.0**

**Table 3:** Outcome of the admitted patients

Outcome	Total	Percent (%)
Discharged	2062	90.5
Referred Out	33	1.5
DAMA	53	2.3
Died	130	5.7
**TOTAL**	2278	100.0

DAMA: discharged against medical advice

## Discussion

The rate of hospital admission for children in this study was high compared to previous studies from both government [2,3,7,11,16] and mission [8,17,18] hospitals. This could be a reflection of the trust most people in the community repose on private hospitals, where they get more personal health attention, and with less protocols to go through compared to government hospitals. Again, Living Word Mission hospital also has a consultant paediatrician which boosts their image as a safe place to manage sick children. The recurrent industrial actions at the government hospitals in recent years eventually benefit private hospitals by increasing the patient loads of the later. The male preponderance in this study is in keeping with previous studies done in other mission hospitals [8,17] and also in the government health settings [10,11,16]. Families preferring to take care of their male children in Africa, compared to their female children is a documented observation in the literature [19]. That more than 90% of the study population were U-5s reflects the vulnerability of this age group to different medical ailments. This is in agreement with the findings of past studies [1,10,11]. This calls for more commitment to the practice of child survival strategies that prevent common childhood diseases, eg National program on immunization, roll back malaria and exclusive breastfeeding etc. Severe malaria as the leading cause of admission is well documented in other studies [1,11]. The battle against malaria in the sub-region has been a long one and it can still be won if the Roll Back Malaria Program can be reactivated and intensified. Severe malaria followed by septicemia, diarrhoea and bronchopneumonia was the same finding in a similar study done at a nearby teaching hospital in Aba [1]. Malaria and infections were also the leading causes of admission in a mission hospital at Owerri, Nigeria [17]. These are preventable medical conditions and reducing their prevalence will require more health education to the masses on scientifically proven, culturally acceptable and family friendly disease prevention strategies.

The mortality rate in this study is comparable to the 6.5% from another mission hospital in Enugu [8], Nigeria. Paradoxically, the mortality rate from these two private mission hospitals are lower than the 9.6% , 11.1%, 12.6%, 9.5%, 9.9%, 10.0%, 14.3% and 15.1% from government hospitals in Aba [1], Lagos [13], Shagamu [20], Ibadan [7], Zaria [21], Ebonyi [22], Lagos [23] and Zaria [24] respectively. It has been observed that patients' satisfactions are more with private hospitals compared with government hospitals [25]. This observation may be attributed to better dedication and commitment of private hospitals' employees compared to those of government establishments. Personal attention given to patients tend to be more “patient friendly” at private hospitals compared to most of our government hospitals [25]. Bad attitude of health care givers can impact negatively on patients' outcome. Again, the extent to which the long waiting periods and “bottle necks” at our government hospitals affect patients' outcome is beyond the scope of this study. Government owned health institutions have more health care specialists than private hospitals and it is expected that patients' outcome should be better in the former. Anything to the contrary is highly unacceptable. The role of the government in ensuring that its health institutions meet up to people's expectation in delivering health care cannot be over-emphasized. More than 90% of these deaths occurred in the U-5s, which is the same finding in most studies from the African sub region [1,9,16,22]. The need for U-5s welfare clinics in all hospitals cannot be overemphasized. Again a more aggressive approach to the management of any admitted child aged less than 5 years should be advocated in our paediatric settings, in view of the vulnerability of this age group to morbidity and mortality. More deaths among males compared to females was also observed in some previous studies [1,9,22]. The differences in immune response places males at a higher risk to morbidity and mortality throughout life, compared to females [26,27].

Septicaemia was the leading cause of death in our study, seconded by severe malaria, and then pneumonia and diarrhoea. This agrees with previous observations [10, 11, 22]. Septicaemia as a leading cause of death may be due to late diagnosis, lack of enough diagnostic tools in our hospital and low index of suspicion for septiceamia. Most cases of septicemia may have been misdiagnosed for malaria initially and managed as such until further investigations confirm septicaemia far into the illness. Symptoms of severe malaria can mimic those of septicaemia, therefore physicians should have a higher index of suspicion for septicaemia whenever they think of malaria. Septicaemia, malaria, pneumonia and diarrhea are all preventable diseases, unlike in the western world where non-communicable diseases tend to be the prevalent causes of mortality in children. The DAMA rate of 2.3% in this study is lower than that observed at some government hospitals in Nigeria [22, 28,29] and outside Nigeria [29-32]. There is paucity of data on DAMA in Private or Mission hospitals globally. A private hospital in India documented a higher DAMA rate of 3.8% [18]. However, the Indian study had a study population consisting of both adults and children that attended the emergency room of the hospital. Our current DAMA rate may be attributed to some of the reasons given by parents who insist on DAMA. These include [18,31,32 ]: financial constraints, domestic obligations, inconvenience of hospitalization, perception that the child is well enough to leave the hospital and extended length of stay in the hospital.

## Conclusion

The knowledge of the pattern of childhood morbidity and mortality in the private health institutions are valuable sources of information which will aid health policy making and intervention strategies. The under-five population remains the more vulnerable group to both childhood morbidity and mortality. Septicaemia, malaria, pnuemonia and acute watery diarrhoea are still the leading causes of morbidity and mortality. Childhood mortality in the mission hospital is lower than that observed in most government hospitals in Nigeria.

### What is known about this topic

- Childhood mortality is still high, especially in the African sub-region;
- The under- fives are the most vulnerable age group to both childhood morbidity and mortality;
- Infections are the commonest causes of morbidity and mortality in African children.

### What this study adds

- Significant number of children in Nigeria are presented to private hospitals by their caregivers when they are sick and rather shy away from government hospitals with more equipment and medical expertise; this calls for more effort from the health workers in the government hospitals to make our government health facilities more “patient friendly”; Government policies should look more into staff dedication and commitment to duties in government hospitals;
- Septicaemia is a leading cause of mortality in children: paediatricians should have a higher index of suspicion for the diagnosis of septicaemia, so that treatment is started early enough to avert mortality;
- The childhood mortality rate was observed to be lower in private hospitals compared to most government hospitals in Nigeria; future researches should be done to find out the reason for such observation and policies need to be formulated to improve patients' outcome in government hospitals.

## Competing interests

The authors declare no competing interest.
